# Melanoma in people living with HIV: antigen-presentation loss, spatial immune escape, and inclusive immunotherapy trials

**DOI:** 10.3389/fimmu.2026.1921391

**Published:** 2026-09-09

**Authors:** Alessio Giubellino, Adrian P. Mansini

**Affiliations:** 1 Department of Laboratory Medicine and Pathology, University of Minnesota, Minneapolis, MN, United States; 2 Department of Dermatology, Rush University Medical Center, Chicago, IL, United States

**Keywords:** antigen presentation, B2M, HLA, immune checkpoint inhibitors, melanoma, people living with HIV, spatial transcriptomics

## Abstract

**Background:**

People living with HIV (PLWH) experience a growing burden of non-AIDS-defining malignancies in the era of effective antiretroviral therapy. Melanoma is an informative but understudied example because its progression and response to immune checkpoint inhibitors are shaped by host immunity and tumor antigen presentation. Yet PLWH have often been excluded from, or only conditionally included in, immunotherapy trials, leaving limited evidence to guide care.

**Scope:**

Emerging clinical and translational studies suggest that melanoma in PLWH may differ from melanoma in HIV-negative patients not only in outcomes and treatment access, but also in antitumor immune organization. This Perspective proposes a spatial immuno-oncology framework in which chronic immune perturbation, impaired antigen presentation, exhausted T-cell states, and suppressive myeloid programs may converge in candidate tumor regions that are less visible to adaptive immunity.

**Implications:**

We highlight the need for batch-balanced spatial profiling cohorts, validation of antigen-presentation pathways at the protein level, prospective immunotherapy studies on tissue samples, and trial eligibility criteria that safely include PLWH.

## Introduction

Melanoma in people living with HIV (PLWH) has often been approached as cancer arising in an immunocompromised host. That framing is incomplete. In the antiretroviral therapy era, PLWH are living longer, and the cancer burden has shifted increasingly toward non-AIDS-defining malignancies (1, 2). Cancer outcomes in PLWH are influenced by immune suppression, chronic inflammation, viral and nonviral comorbidities, differential screening, treatment delays, unequal access to specialized oncology care, and historical exclusion from immune checkpoint inhibitor trials (1–3). Melanoma is informative here because its natural history and therapeutic response depend on tumor-immune recognition, making the relevant question not simply whether it occurs more often in PLWH, but whether it behaves differently once it does.

Epidemiologic studies have produced mixed estimates of melanoma incidence, with some reporting increased risk and others finding no clear independent association after adjustment for established risk factors such as age, ultraviolet exposure, and skin phenotype (4, 5). Older clinical series suggested a more aggressive melanoma course in patients with HIV, including shorter disease-free and overall survival compared with matched HIV-negative controls (6). More recent population-level and immunologic analyses have reinforced concerns about worse outcomes, delayed ICI initiation, and a more immunosuppressive tumor microenvironment in PLWH with melanoma (7).

This Perspective reframes melanoma in PLWH as a spatial immuno-oncology problem. The central hypothesis is that chronic HIV-associated immune perturbation may reshape the melanoma tumor microenvironment in ways that impair antigen presentation, promote exhausted or ineffective T-cell states, expand suppressive myeloid programs, and generate candidate spatial niches of immune escape (7). This model does not imply that HIV status alone determines melanoma biology. Rather, it proposes that HIV control, antiretroviral therapy history, CD4 recovery, viral suppression, inflammation, comorbidities, and access to care should be integrated into tissue-based and clinical studies of melanoma immunity.

## Clinical disparity and biological uncertainty

The clinical literature supports a cautious but important conclusion: melanoma in PLWH is clinically consequential, but the mechanisms underlying outcome differences remain incompletely defined. The early survival data and mixed incidence estimates noted above must be interpreted carefully: older cohorts reporting worse survival were small, often male-predominant, and drawn from earlier HIV treatment eras, while contemporary incidence studies have applied more rigorous adjustment for established risk factors (4–6).

This uncertainty clarifies rather than weakens the rationale for research. Incidence, clinical behavior, and treatment response may be governed by different biology. A modest or inconsistent effect of HIV on melanoma incidence would not exclude a stronger effect on tumor immune contexture, recurrence risk, metastatic spread, or ICI response (7). Because melanoma is among the cancers most dependent on immune recognition, subtle shifts in antigen presentation, T-cell function, myeloid suppression, or spatial immune organization could have clinically meaningful consequences.

Recent work has moved the field beyond epidemiology. A population and immunologic analysis of melanoma in PLWH reported worse long-term outcomes and immune features consistent with impaired antitumor immunity, including checkpoint upregulation, reduced MHC-I antigen-presentation components, including B2M/HLA-I, reduced MHC-II–associated markers, including HLA-DR/CD74, exhausted CD8+ T-cell states, and accumulation of immunosuppressive myeloid-derived suppressor cell (MDSC)-like populations (7). These observations provide a biological framework for why melanoma in PLWH may behave differently even during modern HIV care. The main clinical, biological, and translational domains are summarized in **Supplementary Table 1**.

## Antigen presentation as a central vulnerability

Antigen presentation is a logical axis around which to organize future studies of melanoma in PLWH. Effective CD8+ T-cell recognition requires tumor antigen processing and presentation through MHC class I molecules. In melanoma, defects in this pathway are well-established mechanisms of immune escape and ICI resistance. Loss or alteration of β-2 microglobulin (B2M), HLA class I components, interferon signaling, or antigen-processing machinery can impair tumor recognition by cytotoxic T cells and blunt response to checkpoint blockade (8–11).

This biology is directly relevant to PLWH. Chronic HIV infection is associated with persistent immune activation, altered T-cell differentiation, immune exhaustion, and incomplete immune restoration in some patients despite antiretroviral therapy (12). In melanoma, these alterations in the host may intersect with tumor-intrinsic antigen presentation programs. A tumor with reduced B2M or HLA expression arising in a host with exhausted T cells and expanded suppressive myeloid cells may be less visible to adaptive immunity before treatment begins (7). This does not mean that PLWH are intrinsically poor candidates for ICI; rather, the biology of response and resistance should be measured, not assumed.

Reduced antigen presentation may also connect tissue biology with spatial architecture. A decrease in B2M, HLA class I, HLA class II, CD74, TAPBP, or antigen-processing proteases may have different biological implications depending on where it occurs (8, 9). Loss at the invasive margin, where melanoma cells interact with stromal and immune barriers, could be relevant to local invasion, immune exclusion, and metastatic escape (13). Reduced HLA class II or CD74 in antigen-presenting cell-rich regions could reflect impaired local priming or altered macrophage and dendritic-cell function. These questions require spatially resolved approaches (14).

## Why spatial biology matters

Bulk transcriptomics and conventional pathology each capture only part of the melanoma immune landscape. Bulk profiling can identify immune pathways but loses anatomic context. Histology can classify tumor infiltrating lymphocytes (TILs) as brisk, non-brisk, or absent, but cannot resolve molecular states across tumor and immune compartments. Single-cell RNA sequencing defines cell states, but tissue dissociation removes the spatial relationships that determine whether immune cells can recognize, contact, and kill melanoma cells. Spatial profiling bridges these gaps.

Spatial context is clinically relevant in melanoma immunotherapy. Pre-existing CD8+ T cells at the invasive tumor margin have been associated with response to programmed cell death protein 1 (PD-1) blockade, supporting the concept that the geography of antitumor immunity is meaningful (13). Spatial technologies, such as digital spatial profiling, now allow RNA and protein programs to be measured from morphologically defined tumor, immune, stromal, epidermal, and invasive-edge compartments in archival formalin-fixed paraffin-embedded (FFPE) tissues (14). This approach is valuable for PLWH, where prospective tissue collections are limited and historical cohorts may be available only as archived pathology specimens.

Spatial profiling should also be interpreted together with clonal information from the T-cell receptor repertoire. The presence of lymphocytes within or around melanoma does not necessarily indicate tumor-directed immunity; infiltrates may be nonspecific, bystander, exhausted, or spatially disconnected from antigen-presenting tumor regions. Pairing spatial maps with T-cell receptor diversity and clonality could help distinguish diffuse inflammation from expanded T-cell populations potentially responding to melanoma antigens. In PLWH, this distinction may be particularly important because chronic viral antigen exposure and systemic immune perturbation could shape local T-cell phenotypes independently of tumor recognition. Therefore, future melanoma-HIV studies should consider integrating spatial profiling with TCR sequencing, CD8+ T-cell functional markers, and proximity-based analyses of tumor-cell contact (13, 15–17).

A spatial framework also reduces oversimplification. Melanoma in PLWH should not be reduced to “less immunity.” A tumor may contain immune cells that are spatially excluded from melanoma nests (18, 19), concentrated at the margin, trapped in stromal regions, functionally exhausted, or disconnected from antigen-presenting compartments. Tumor-intrinsic resistance programs may also contribute to T-cell exclusion and resistance to checkpoint blockade, supporting the need to analyze spatial immune organization alongside melanoma-cell states (18, 19). Conversely, reduced immune gene expression may reflect technical batch effects, sampling differences, tissue age, cellular composition, or true biological immune suppression. Spatial studies must therefore use batch-balanced cases and controls, patient-level modeling, matched clinical covariates, and orthogonal protein validation. In this context, spatial immune escape should be treated as a spatially resolved research hypothesis that requires validation across independent cohorts rather than as a settled biological feature of melanoma in PLWH. Testing that hypothesis at scale, however, depends on access to PLWH tissue and clinical data that current trial structures rarely generate.

Because the melanoma-HIV spatial immune-escape framework is currently informed by limited direct tissue-based evidence, including one recent population-immunology study, independent validation in batch-balanced, clinically annotated cohorts is essential before these features are treated as established biological properties of melanoma in PLWH (7).

## From exclusion to evidence generation

The exclusion of PLWH from ICI trials has created a circular problem: patients are excluded because evidence is limited, and evidence remains limited because patients are excluded. This is especially problematic in melanoma, where ICI, particularly PD-1–based therapy, is central to standard-of-care treatment for advanced disease (20). A systematic analysis of cancer ICI trials found that many excluded PLWH or allowed enrollment only conditionally (3). This practice has persisted despite growing clinical evidence that ICI can be administered safely to selected PLWH, particularly those receiving antiretroviral therapy with controlled viral load (21, 22).

Regulatory guidance has moved in a more inclusive direction. FDA recommendations state that patients with HIV should be considered for cancer trial inclusion based on immune function, HIV control, potential drug interactions, and the scientific rationale of the study rather than excluded categorically (23). For melanoma trials, this principle translates into study-appropriate CD4 criteria, viral-load control requirements, antiretroviral therapy stability, opportunistic infection history, and drug-drug interaction monitoring.

Inclusive trial design should also be biologically informative. PLWH enrolled in melanoma trials should not merely be counted as a subgroup. Their tumors should be linked to tissue, blood, and clinical endpoints that can define mechanisms of response and resistance. Baseline biopsies, on-treatment biopsies when feasible, circulating immune profiling, HIV viral load, CD4/CD8 ratio, antiretroviral regimen, and immune-related adverse events should be collected systematically. This would allow the field to determine whether antigen-presentation loss, exhausted T-cell states, myeloid suppression, or spatial immune exclusion predict ICI outcomes in PLWH (7, 9). A practical research agenda for tissue-linked melanoma-HIV studies is summarized in **Table 1**.

**Table 1:** Research agenda for a tissue-linked melanoma and HIV program.

Priority	Study design	Minimum assays	Key endpoints
1. Validate antigen-presentation loss	Batch-balanced retrospective FFPE cohort of melanoma from PLWH and HIV-negative controls	IHC/multiplex IF for B2M, HLA class I, HLA-DR, CD74, CD8, CD45, CD68/CD163	Protein-level antigen-presentation score; tumor versus immune compartment localization
2. Test spatial immune escape as a candidate mechanism	GeoMx, Visium, Xenium, CosMx, or multiplex IF in matched primary and metastatic tumors	Tumor, immune, stromal, epidermal, and invasive-edge compartment profiling	CD8-tumor proximity; candidate antigen-presentation-low tumor niches, defined by reduced B2M/HLA-I, reduced HLA-DR/CD74, or broader antigen-processing machinery-low regions; myeloid-rich suppressive regions
3. Link tissue biology to ICI response	Prospective or retrospective ICI-treated melanoma cohort including PLWH	Pretreatment tissue, blood immune phenotyping, HIV viral load, CD4 count, ART regimen	Response rate, progression-free survival, overall survival, immune-related adverse events
4. Define HIV-specific modifiers	Stratified analysis among PLWH	CD4 count, CD4/CD8 ratio, nadir CD4, viral suppression, ART class, coinfections	Association between HIV control and tumor immune phenotype
5. Improve trial inclusion	Embedded PLWH cohort within melanoma ICI trials	Standard safety labs, HIV monitoring, immune profiling	Feasibility, safety, efficacy, and biomarker-defined benefit
6. Identify therapeutic strategies beyond MHC-I dependence	Translational studies of antigen-presentation-low melanoma, stratified by B2M/HLA-I loss, HLA-DR/CD74 reduction, and broader antigen-processing machinery defects	NK-cell markers, stress ligands, macrophage phagocytosis markers, bispecific or innate immune targets	Candidate MHC-dependent and MHC-independent strategies for antigen-presentation-deficient tumors

ART, antiretroviral therapy; B2M, beta-2 microglobulin; CD, cluster of differentiation; CD74, cluster of differentiation 74; FFPE, formalin-fixed paraffin-embedded; HIV, human immunodeficiency virus; HLA, human leukocyte antigen; HLA-I, human leukocyte antigen class I; HLA-DR, human leukocyte antigen DR isotype; ICI, immune checkpoint inhibitor; IF, immunofluorescence; IHC, immunohistochemistry; MHC, major histocompatibility complex; MHC-I, major histocompatibility complex class I; NK, natural killer; PLWH, people living with HIV.

In this table, antigen-presentation-low refers to reduced B2M/HLA-I expression, reduced HLA-DR/CD74 expression, or broader defects in antigen-processing machinery, depending on the tumor and immune compartment analyzed.

**Figure 1:**
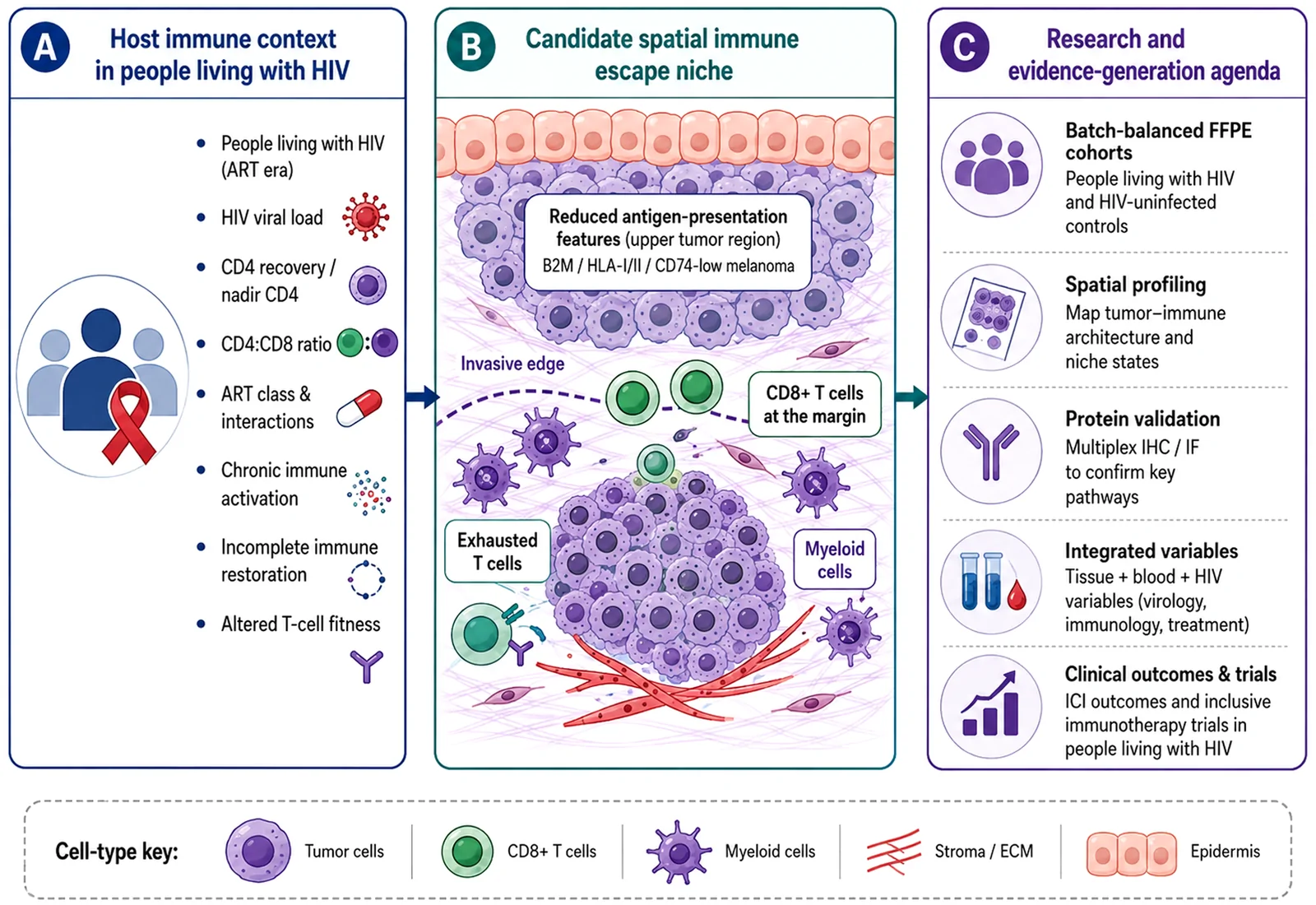
Spatial immuno-oncology framework for melanoma in people living with HIV. The proposed model links HIV-associated immune perturbation with altered melanoma tumor-immune organization and nominates spatial immune escape as a candidate mechanism for future study. Chronic immune activation, incomplete immune restoration, altered CD4/CD8 balance, and antiretroviral therapy-related clinical variables may intersect with melanoma antigen-presentation programs. Candidate tumor regions with reduced B2M, HLA class I/II, or CD74 expression may be less visible to adaptive immunity and may coexist with exhausted CD8+ T-cell states, suppressive myeloid programs, and altered immune positioning at tumor-stromal interfaces, including the invasive edge. The cell-type key identifies tumor cells, CD8+ T cells, myeloid cells, stroma/extracellular matrix, and epidermis. This framework supports batch-balanced FFPE cohorts, spatial profiling, protein-level validation, tissue-linked immune checkpoint inhibitor outcome studies, and inclusive melanoma immunotherapy trials that prospectively collect HIV-specific clinical and immune parameters. ART, antiretroviral therapy; B2M, beta-2 microglobulin; CD, cluster of differentiation; CD8, cluster of differentiation 8; CD74, cluster of differentiation 74; ECM, extracellular matrix; FFPE, formalin-fixed paraffin-embedded; HIV, human immunodeficiency virus; HLA-I/II, human leukocyte antigen class I/class II; ICI, immune checkpoint inhibitor; IF, immunofluorescence; IHC, immunohistochemistry; PLWH, people living with HIV.

This conceptual spatial immuno-oncology framework is illustrated in **Figure 1**.

## A practical model for future studies

Future melanoma-HIV studies should move beyond descriptive, region-level comparisons and prioritize designs that put the single patient at the center. Multiple tumor regions should be analyzed in the same histologic section, batch effects should be corrected across HIV status, and clinical variables should be captured systematically. HIV status should be annotated together with CD4 count, nadir CD4 when available, HIV viral load, antiretroviral therapy, coinfections, smoking history, ultraviolet risk proxies, tumor stage, Breslow thickness, ulceration, tumor-infiltrating lymphocyte (TIL) grade, treatment timing, and immunotherapy exposure. This design would allow spatial immune phenotypes to be interpreted as biological signals rather than technical or sampling artifacts.

The first translational priority should be protein-level validation of antigen presentation. B2M and HLA class I are essential because they directly determine whether melanoma cells can present antigen to CD8+ T cells (8, 9). HLA-DR and CD74 are also important because MHC class II expression in melanoma and antigen-presenting compartments may influence local immune activation and response to immunotherapy (24). These markers should be quantified separately in melanoma cells, immune cells, and stromal regions, with attention to the invasive margin. A minimal immune-spatial panel should include a melanoma-cell marker such as SOX10, Melan-A/MART-1, or PMEL/HMB45, together with CD8, PD-1, LAG-3, CD68, CD163, HLA-DR, CD11b, and CD33.

Longitudinal sampling should be prioritized whenever clinically feasible. Baseline tissue, early on-treatment biopsies, and progression or relapse specimens could help determine whether antigen-presentation loss, CD8+ T-cell exclusion, T-cell exhaustion, and myeloid suppression are pre-existing features or acquired mechanisms of resistance during ICI therapy. This temporal design would be especially informative in PLWH, where host immune status, viral suppression, CD4 recovery, and antiretroviral therapy may change over time and may interact with tumor-intrinsic immune escape. Paired tissue analysis could also clarify whether B2M/HLA-I loss, HLA-DR/CD74 reduction, or broader antigen-processing defects emerge under therapeutic pressure or mark tumors that are less visible to adaptive immunity before treatment begins (8, 9, 15).

The second priority is integration with clinical outcome. Antigen-presentation loss is most meaningful if it predicts recurrence, metastasis, lack of response to ICI, early progression, or immune-related toxicity. Rather than treating HIV status itself as a predictor of ICI benefit (21), studies should ask which PLWH have tumors that remain immunologically visible and which have tumors that require alternative strategies, such as approaches that enhance antigen presentation, engage innate immunity, target suppressive myeloid compartments, or exploit MHC-independent mechanisms.

The third priority is inclusion in clinical trials. Future melanoma trials should presumptively include PLWH when HIV is controlled and safety criteria are met (3, 23). Trials should collect HIV-specific parameters prospectively, including viral load, CD4 count, antiretroviral therapy, opportunistic infection history, and drug-drug interactions. Doing so would allow inclusion to generate knowledge rather than merely broaden eligibility.

## Therapeutic implications beyond MHC-I-dependent immunity

If antigen-presentation loss proves to be enriched or clinically meaningful in melanoma from PLWH, future studies should not focus exclusively on conventional MHC-I-dependent T-cell reinvigoration. Reduced B2M/HLA-I expression may limit CD8+ T-cell recognition even when checkpoint pathways are blocked, making MHC-independent or innate immune strategies conceptually important. These could include NK-cell–directed approaches, such as NKG2A or KIR blockade and NK-cell engagers; intratumoral immune activation with oncolytic viruses; CD40 agonists or other innate immune activators; and bispecific strategies that redirect immune effector cells toward melanoma-associated surface targets. LAG-3 inhibition is also relevant because it may address dysfunctional or exhausted T-cell states without assuming that all resistance is solely PD-1 dependent. These strategies should not be presented as established therapies for melanoma in PLWH, but as rational translational directions to test in antigen-presentation-low tumors using tissue-linked trials (24–28).

## Discussion

Melanoma in PLWH sits at the intersection of cancer immunology, spatial biology, infectious disease, and therapeutic equity. The key unanswered question is not simply whether HIV increases melanoma incidence, but whether melanoma arising in PLWH occupies a tumor-immune state that affects immune surveillance, metastatic progression, and response to checkpoint blockade. Antigen-presentation loss provides a compelling axis for study because it is measurable, spatially interpretable, clinically relevant, and mechanistically linked to immune escape. Spatial profiling can test whether this loss is regionally organized, which immune cells surround these regions, and whether such patterns identify tumors capable of escaping adaptive immune control. A tissue-linked, spatially resolved, and clinically inclusive research agenda could identify biomarkers of resistance, improve treatment selection, and ensure that advances in melanoma immunotherapy benefit PLWH rather than leaving them at the margins of evidence generation.

## Data Availability

The datasets presented in this study can be found in online repositories. The names of the repository/repositories and accession number(s) can be found in the article/**Supplementary Material**.
