# Supplementary material for: Melanoma in people living with HIV: antigen-presentation loss, spatial immune escape, and inclusive immunotherapy trials
Source: Front Immunol. 2026 Sep 9;17:1921391. doi: 10.3389/fimmu.2026.1921391 (PMC13597333; doi:10.3389/fimmu.2026.1921391)
Supplement: Supplementary file 1 [file Table1.docx]

**Supplementary Table 1. Proposed evidence framework for melanoma in PLWH**

| **Domain** | **What is known** | **Key uncertainty** | **Research implication** |
| --- | --- | --- | --- |
| **Epidemiology** | Some studies suggest increased melanoma risk in PLWH, while others find no clear independent association after adjustment for risk factors (4,5). | Whether HIV independently increases melanoma incidence remains unresolved. | Separate incidence questions from progression, immune escape, and treatment-response questions. |
| **Clinical outcomes** | Older matched series and newer population analyses suggest worse outcomes in PLWH with melanoma (6,7). | How much is driven by biology versus delayed diagnosis, treatment access, comorbidities, or trial exclusion? | Use prospective cohorts with stage, treatment timing, HIV control, and tissue-linked biomarkers. |
| **Antigen presentation** | B2M/HLA loss and related antigen-presentation or interferon-pathway alterations are known mechanisms of melanoma immune escape and ICI resistance (8–11). | Whether antigen-presentation loss is enriched in melanoma from PLWH and whether it predicts ICI outcomes. | Validate B2M, HLA class I, HLA-DR, CD74, TAPBP, and antigen-processing markers by IHC or multiplex IF. |
| **T-cell dysfunction** | Recent melanoma studies in PLWH report exhausted CD8+ T-cell features and checkpoint enrichment (7). | Whether these states are tumor-specific, HIV-associated, treatment-related, or shaped by local tissue niches. | Combine spatial profiling with T-cell phenotyping, TCR analysis, and clinical response data. |
| **Myeloid suppression** | MDSC-like and immunosuppressive myeloid populations may be enriched in melanoma from PLWH (7). | Whether these cells directly mediate ICI resistance or reflect systemic inflammation. | Profile CD11b, CD33, HLA-DR, CD68, CD163, ARG1, IL10, and proximity to tumor/CD8 compartments. |
| **Trial inclusion** | PLWH have historically been excluded from many ICI trials (3), despite emerging safety data supporting ICI use in selected patients with HIV and cancer (21, 22). | Whether eligibility criteria are still more restrictive than clinically justified. | Align melanoma trials with modern FDA guidance and collect HIV-specific safety and efficacy endpoints (23). |

**Abbreviations:** ARG1, arginase 1; B2M, beta-2 microglobulin; CD, cluster of differentiation; FDA, U.S. Food and Drug Administration; HIV, human immunodeficiency virus; HLA, human leukocyte antigen; HLA-DR, human leukocyte antigen DR isotype; ICI, immune checkpoint inhibitor; IF, immunofluorescence; IHC, immunohistochemistry; IL10, interleukin 10; MDSC, myeloid-derived suppressor cell; PLWH, people living with HIV; TCR, T-cell receptor.
